# Common Data Elements for Disorders of Consciousness: Recommendations from the Working Group on Goals-of-care and Family/Surrogate Decision-Maker Data

**DOI:** 10.21203/rs.3.rs-3084539/v1

**Published:** 2023-06-26

**Authors:** Matthew N. Jaffa, Hannah L. Kirsch, Claire J. Creutzfeldt, Mary Guanci, David Y. Hwang, Darlene LeTavec, Dea Mahanes, Alexis Steinberg, Girija Natarajan, Darin B. Zahuranec, Susanne Muehlschlegel

**Affiliations:** Hartford Hospital; Stanford University School of Medicine; University of Washington School of Medicine; Massachusetts General Hospital; The University of North Carolina at Chapel Hill School of Medicine; None (family representative); UVA Health; University of Pittsburgh School of Medicine; Children’s Hospital of Michigan; University of Michigan; University of Massachusetts Chan Medical School

**Keywords:** coma, consciousness, common data elements, goals of care, surrogate decision-maker, shared decision-making

## Abstract

**Introduction:**

In order to facilitate comparative research, it is essential for the fields of neurocritical care and rehabilitation to establish common data elements (CDE) for disorders of consciousness (DoC). Our objective was to identify CDEs related to goals-of-care decisions and family/surrogate decision-making for patients with DoC.

**Methods:**

To achieve this, we formed nine CDE working groups as part of the Neurocritical Care Society’s Curing Coma Campaign. Our working group focused on goals-of-care decisions and family/surrogate decision-makers created five subgroups: (1) clinical variables of surrogates, (2) psychological distress of surrogates, (3) decision-making quality, (4) quality of communication, and (5) quality of end-of-life care. Each subgroup searched for existing relevant CDEs in the NIH/CDE catalog and conducted an extensive literature search for additional relevant study instruments to be recommended. We classified each CDE according to the standard definitions of “core,” “basic,” “exploratory,” or “supplemental,” as well as their utility for studying the acute or chronic phase of DoC, or both.

**Results:**

We identified 32 relevant pre-existing NIH CDEs across all subgroups. A total of 34 new instruments were added across all subgroups. Only one CDE was recommended as disease core, the “mode of death” of the patient from the clinical variables subgroup.

**Conclusions:**

Our findings provide valuable CDEs specific to goals-of-care decisions and family/surrogate decision-making for patients with DoC that can be used to standardize studies to generate high-quality and reproducible research in this area.

## Introduction

In 2019, the Neurocritical Care Society launched the Curing Coma Campaign to bring together clinician and non-clinician scientists in a common scientific goal: to identify the gaps in our understanding of disorders of consciousness (DoC) and develop new research across the full spectrum of care.^1^ Three pillars were identified as the fundamental components needed to bring the science and care of patients with DoC forward. These pillars are (1) endotyping to better characterize coma and its clinical trajectory, (2) discovering biomarkers of coma and coma recovery, and (3) conducting proof-of-concept clinical trials to inform and advance the design of future trials. From this work, it became clear that the development of well-defined common data elements (CDE) would be paramount to the success of future scientific work in this area.^2^

CDEs are standardized data points and instruments that enhance reproducibility and data comparability for research trials to facilitate disease-specific scientific communication and progress^3^. The National Institute of Neurological Disorders and Stroke (NINDS) has led the development of CDEs for multiple neurological diseases and disorders since 2005 with the goal of improving data consistency among NINDS-funded projects.

For the Curing Coma Campaign’s DoC CDE project, nine working groups were created, including one focused goals-of-care, family/surrogate decision-maker data (F/SDM) and related outcomes. This focus is novel for a CDE project, as there are no prior CDE projects addressing data elements related to surrogate medical decision making, or surrogate psychological outcomes or well-being. With increasing emphasis in critical care on the best ways to communicate with surrogates of patients with severe acute brain injury and facilitate high-quality decision-making, it is imperative that the neurocritical care field develop consistent and uniform instruments that may facilitate research on these aspects of clinical care.^4,5^ The goal of this working group was to develop CDEs for goals-of-care and F/SDM data. CDEs like those proposed by our working group will enable the community to compare family and surrogate interventions, as we as a medical community continue to explore this understudied aspect of neurocritical care.

## Methods

The Steering Committee for the DoC CDE project identified leaders with expertise in each of the identified task groups: clinical/behavioral phenotyping, hospital course/confounders, imaging, electrophysiology, biospecimens, physiologic data (“big data”), therapeutic interventions, outcomes, and endpoints, and goals-of-care and F/SDM data. The chair of each working group subsequently identified and invited members from across the Neurocritical Care Society, including trainees in fellowship, junior faculty members, non-physician clinicians (e.g. nurses, pharmacists, advanced practice providers), senior physician content experts, and at least one family advocate. Each working group then drafted a charter to describe its mission and scope, including the process overview, reporting plan, and deliverables.

**Figure 1:**
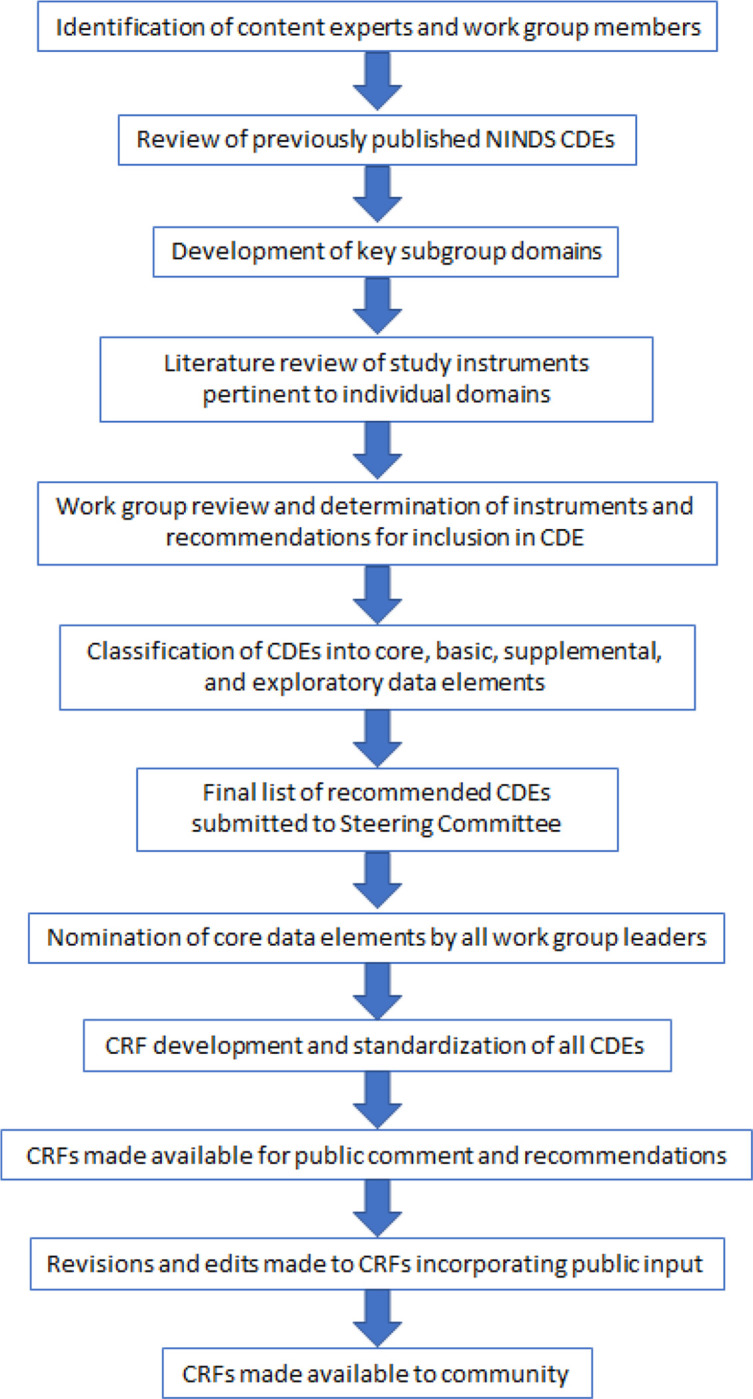
Development process of the goals-of-care and family/surrogate decision maker CDEs Outline of our group’s work process for the review of existing CDEs and addition of new CDEs for inclusion in our goals-of-care and family/surrogate decision maker CDEs

Work groups were tasked with identifying and/or developing CDEs applicable to their mission and scope. Our group’s work process is described in Fig. 1. First, the working group performed an in-depth review of the existing CDEs published at https://www.commondataelements.ninds.nih.gov for other neurological diseases pertinent to the overall scope of goals-of-care decisions and the impact of decision-making on F/SDM of patients with DoC.

Early meetings of the working group focused on the development of several key domains important to the care of surrogate decision makers of patients with DoC. These domains included (1) clinical variables of F/SDM (i.e., family/surrogates’ relevant demographic information), (2) psychological well-being of F/SDM, (3) decision making quality, (4) quality of communication, and (5) quality of end-of-life care. For each subgroup, we identified 2–3 co-authors who were tasked with conducting a literature review to identify any known study instruments relevant to their domain. Subgroups also determined through a literature search whether or not instruments had been previously validated in populations of patients with DoC or other relevant neurological disorders. All questionnaires requiring patient participation were excluded, as the working group was intended to focus on goals-of-care and F/SDM outcomes and as this patient population is typically not able to participate in surveys and similar instruments due to their DoC. Following review by individual subgroups, the full working group met to discuss each CDE and scale to determine its relevance to DoC and the working group’s scope. Over several meetings, the list of potential relevant CDEs was narrowed, and subsequent meetings focused on classifying each study instrument as described below.

To maintain consistency with previously published CDEs, each working group was responsible for classifying each of the recommended CDEs and instruments as “Core”, “Basic,” “Supplemental,” or “Exploratory,” according to the following standard definitions:^6^

1. Core CDEs—elements which should be consistently collected across all studies of DoC and which are required for collection in all studies of DoC regardless of neurological disease and study focus;
2. Basic CDEs—elements that are essential based on certain conditions or study focus and that are strongly recommended for studies related to these conditions or study foci in DoC;
3. Supplemental CDEs—elements that are commonly recommended for specific DoC studies, depending on the context and goals of the specific study;
4. Exploratory CDEs—elements which are reasonable to use, but whose validity is limited due to insufficient availability of data; thus, they require further validation.

We further classified CDEs according to their utility in the acute phase of DoC (the initial admission for the inciting neurological event through hospital discharge), the chronic phase (post-discharge DoC, including patients in rehabilitation centers, nursing facilities, or home), or both. A final list of CDEs was submitted to the Steering Committee. Work group leaders met to nominate and agree upon the Disease Core CDEs. Case report forms (CRF) for each CDE were developed by each working group and made available for public comment prior to finalizing the CRFs with inclusion of public feedback. All finalized CRFs are available to the scientific community focused on studying patients with DoC at https://zenodo.org/record/7210236#.ZCXwb-zML0p.

## Results

Our working group identified five domains.

### Clinical variables

We identified 22 relevant pre-existing NIH CDEs for this subgroup and ten additional items through literature review (Table 1). We also identified “mode of death” (death from withdrawal of life sustaining treatments or brain death or respiratory/cardiac arrest) of the patient as an important clinical variable related to goals-of-care, as surrogates decide on withdrawal of life sustaining treatments during their goals-of-care decision-making process. “Mode of death” of the patient/subject, was identified as a Disease Core CDE. An additional five elements were identified as basic; the remainder were supplemental and exploratory.

No relevant pre-existing NIH CDEs were identified for this subgroup. Five supplemental clinical data elements were added in this domain (Table 2). One, the Decisional Regret Scale, is recommended only for use in the chronic phase of DoC; the others are recommended for use solely in the acute phase of DoC.

### Quality of communication

No relevant pre-existing NIH CDEs were identified for this subgroup. A total of six instruments were added in this domain, three supplemental and three exploratory (Table 3). The Critical Care Family Needs Inventory (CCFNI) is recommended for use only in the acute phase of DoC, and the Modified Caregiver Strain Index (M-CSI) is recommended for use only the chronic phase of DoC.

### Quality of end-of-life care

No relevant pre-existing NIH CDEs were identified for this subgroup. A total of six instruments were identified in this domain. Three were excluded from consideration as they required patient-reported assessments, which is not possible in patients with DoC. Three instruments were chosen for inclusion as supplemental CDEs (Table 4).

### Surrogate psychological well-being

We identified 10 relevant pre-existing NIH CDEs (Table 5). All were deemed supplemental. Two CDEs (the Center for Epidemiologic Studies Depression Scale (CEDS) and Zarit Caregiver Burden Scale) apply only to chronic DoC; we recommend the remainder for use in both acute and chronic DoC. Eight additional instruments were added in this category, all of which are exploratory (Table 5). Of these, the Brief Cope Inventory (BCI), the National Stressful Events Survey PTSD Short Scale (NSESSS), the Modified Caregiver Strain Index (M-CSI), and the Post-Traumatic Stress Disorder Checklist (PCL-5) are recommended for use only in the chronic phase of DoC. The remainder can be used in both the acute and chronic phase of DoC.

## Discussion

As the neuroscience community explores the biological underpinnings of and new treatments for DoC, we must also work to understand the psychosocial impacts on caregivers. Studies have demonstrated a high prevalence of depression and post-traumatic stress disorder in families of patients who died in ICU [81]. However, few thoroughly tested study instruments exist that specifically address decision-making, end-of-life, and caregiver quality of life and psychological outcomes in DoC.

There are many nonmodifiable risk factors for post-ICU mental health challenges in patient surrogates; however, multiple studies have also suggested modifiable factors, including discordance between preferred and actual decision-making roles and passivity in decision-making [82, 83]. These modifiable risk factors indicate an opportunity to better evaluate and prevent post-ICU PTSD, anxiety, and depression in surrogate decision-makers and family members. Proposed interventions to reduce these symptoms of psychological distress have ranged from structured storytelling and sensory awareness training [84] to interprofessional support interventions [85]. In neurocritical care specifically, shared decision-making tools are being explored to address the challenges of decision-making in the setting of uncertain prognosis and to help families arrive at patient goal-concordant decisions in which they feel confident and reduce decisional conflict and decision regret [86–90].

There are no specific surveys or other instruments currently available that have been specifically developed for evaluation of F/SDM outcomes following surrogate decision-making for patients with DoC. The inconsistent use of specific instruments to evaluate the experiences of F/SDM is among the challenges in establishing efficacy and reproducibility of results pertaining to decision tools, family interventions, and serious illness communication techniques. While limited by the lack of extensive work in our specific disease population, our group has collated instruments that are well-validated in comparable populations. The consistent, widespread use of these instruments could promote careful study of communication, decision-making, and end of life management in our study population, and thus improve the care of patients with DoC and their caregivers. This CDE for goals-of-care and F/SDM is among the first necessary steps to harmonize all future research in this special population of caregivers and decision-makers for patients with DoC. Additional next steps include the validation of high-value study instruments specifically in F/SDM of patients with DoC, as well as adapting extant instruments to better address concerns and outcomes pertinent to this population.

## Figures and Tables

**Table 1:** Clinical Variables

CDE/Scale Name	Subgroup	Number of Items/Questions	NINDS CDE ID	Brief Description	Classification
**Mode of death of patient/subject**	Clinical Variables	1		Mode of death: Brain death; cardiorespiratory death; withdrawal of life sustaining treatments	Core
**Family history relative age value of family member**	Clinical Variables	1	C14944	Value for participant’s/subject’s family member’s current age	Basic
**Birth sex assigned type of family member**	Clinical Variables	1	C58676	Self-reported phenotypic sex of participant/subject, assigned at birth.	Basic
**Gender identity type of family member**	Clinical Variables	1	C58677	Gender identity self-identified by the subject/participant	Basic
**Ethnicity category of family member**	Clinical Variables	1	C00020	Category of ethnicity the participant/subject most closely identifies with	Basic
**Race category of family member**	Clinical Variables	1	C00030	The patient’s self declared racial origination, independent of ethnic origination, using OMB approved categories.	Basic
**Language spoken fluent ISO code by family member**	Clinical Variables	1	C00028	Code (ISO 639-2) for each language that the participant/subject speaks fluently	Supplemental
**Religion type of family member**	Clinical Variables	1	C11115	Religion the family member practices or with which he/she identifies.	Supplemental
**Participation Assessment with Recombined Tools-Objective (PART-O) - Religious spiritual service number score for family member**	Clinical Variables	1	C07369	Score for the number of times the family member attends religious or spiritual services in a typical month.	Supplemental
**Religiosity scale for family member**	Clinical Variables	5		Duke University Religion index-5 item instrument to assess organizational and non-organizational religious activity, and intrinsic religiosity	Supplemental
**Single-item religiosity scale for family member**	Clinical Variables	1		Score for rating strength of influence religion has in family member’s life	Supplemental
**Subjective numeracy scale for family member** ^7^	Clinical Variables	7		Self-report measure of perceived ability to perform various mathematical tasks and preferences for the use of numerical versus prose information; found to predict comprehension of risk communications and ability to complete utility elicitations.	Supplemental
**Short Assessment of Health Literacy – Spanish and English (SAHL-S&E)** ^8^	Clinical Variables	18		Test of comprehension of health literacy	Supplemental
**Rapid Estimate of Adult Literacy in Medicine (REALM) for family member** ^9^	Clinical Variables	66		Word-recognition of 66 medical terms	Supplemental
**Family income range of family member**	Clinical Variables	1	C00205	Range, in U.S. dollars, of the annual pre-tax, pre-deduction total income, of the household of which the participant/subject is a member	Supplemental
**Family income supported persons count of family member**	Clinical Variables	1	C00206	Count of all people, including the participant/subject, who are supported by the household gross annual income reported	Supplemental
**Marital or partner status of family member**	Clinical Variables	1	C00207	Status of participant/subject’s current domestic relationship, whether marital or partnered	Supplemental
**Employment fulltime status of family member**	Clinical Variables	1	C10678	Status of participant/subject’s current full-time employment	Supplemental
**Education level type of family member**	Clinical Variables	1	C00012	Highest grade or level of school participant/subject has completed or the highest degree received	Supplemental
**ZIP partial code of family member**	Clinical Variables	1	C10677	First three digits of the zip code where the participant/subject currently lives.	Supplemental
**Family support for family member**	Clinical Variables	1		Person count who are involved in supporting the family member in decision-making	Supplemental
**Comfort care indicator**	Clinical Variables	1	C14279	Indicates if patient’s care restricted to comfort measures only	Supplemental
**Comfort care earliest documentation timepoint**	Clinical Variables	1		Timepoint of earliest documentation of transition to comfort directed measures (CMO) during hospitalization	Supplemental
**Documented treatment preferences**	Clinical Variables	1		Indicates if patient had previously documented treatement preferences	Supplemental
**DNR/DNI indicator of patient**	Clinical Variables	1	C14282	Indicates if patient was made DNR/DNI (Do not resuscitate or Do not intubate) during hospitalization	Supplemental
**DNR/DNI earliest documentation timepoint type of patient/subject**	Clinical Variables	1	C14283	Timepoint of earliest documentation of DNR/DNI (Do not resuscitate or Do not intubate) during hospitalization	Supplemental
**Modified Rankin scale score-premorbid of patient/subject**	Clinical Variables	1	C13230	The overall pre-morbid modified Rankin Scale (mRS) score assigned to the participant/subject	Supplemental
**Single item pre-morbid cognitive status**	Clinical Variables	1		Rating of subject’s memory at the present time by family member on a 5-point scale (1–5) with a higher score indicating worse perceived memory	Supplemental
**Pre-morbid developmental and academic history type of pediatric patient**	Clinical Variables	10	C11084	Specific aspects of the pediatric patient’s pre-morbid developmental status and academic function	Supplemental
**Pre-morbid school placement type of pediatric patient**	Clinical Variables	1	C11086	The description of the type of education the pediatric patient receives with details of being with or without assistance.	Supplemental
**Death location type of patient/subject**	Clinical Variables	1	C12610	Type of location where the participant/subject died	Exploratory
**Palliative care consulted**	Clinical Variables	1		Was palliative care consulted	Exploratory
**Hospice services indicator**	Clinical Variables	1		Did the patient receive formal hospice services.	Exploratory
**Family history medical condition indicator**	Clinical Variables	1	C00721	Indicator of whether a family member or first and second degree blood relatives of the patient has had a history of the particular medical condition or health related event ie. coma	Exploratory
**Family meetings count**	Clinical Variables	1		number of family meetings the family member took part in	Exploratory
**Family meetings duration**	Clinical Variables	1		Were the family meetings with the provider team in person or over phone /video	Exploratory
**Single-item maximizer-minimizer elicitation question (the MM1) of family member**	Clinical Variables	1		valid, brief elicitation of maximizing-minimizing preferences	Exploratory

**Table 2:** Quality of Decision Making

CDE/Scale Name	Subgroup	Number of Items/Questions	NINDS CDE ID	Brief Description	Classification
**Family Satisfaction of ICU care (FS-ICU 24R)** ^10,11^	Decision quality	24		Assessment of family satisfaction related to medical decision making as completed during their ICU stay.	Supplemental
**Decisional conflict scale** ^12^	Decision Quality	16		Measure of personal perception of uncertainty in choosing options, modifiable contributors to uncertainty and effective decision making.	Supplemental
**Decisional regret scale** ^13^	Decision Quality	5		Measures distress or remorse after a healthcare decision.	Supplemental
**Decision SElf Efficacy** ^14^	Decision Quality	11		Measures self-confidence or belief in one’s abilities to make decisions	Supplemental
**Prognostic concordance between clinician and family** ^15–17^	Decision Quality	6		Assessment of how well family understands a doctor’s prognosis	Supplemental

**Table 3:** Communication Quality

CDE/Scale Name	Subgroup	Number of Items/Questions	NINDS CDE ID	Brief Description	Classification
**Critical care family need inventory** ^18–21^	Quality of communication	46		Inventory of specific family needs/preferences in ICU stay	Supplemental
**Family inpatient communication survey** ^22,23^	Quality of communication	30		Experiences of family members with communication on the part of inpatient care team	Supplemental
**Quality of communication** ^24,25^	Quality of communication	19		Assessment of quality of physician communication	Supplemental
**9-item Shared Decision-making Questionnaire (SDM-Q-9)** ^32–39^	Quality of communication	9		Assessment of effectiveness of shared decision-making in-hospital	Exploratory
**CollaboRATE** ^40–41^	Quality of communication	10		Assessment of effectiveness of shared decision-making	Exploratory

**Table 4:** Quality of End-of-Life Care

CDE/Scale Name	Subgroup	Number of Items/Questions	NINDS CDE ID	Brief Description	Classification
**Quality of Dying and Death (QODD)** ^42,43^	Quality of End-of-life Care	17		Questionnaire assessing the overt quality of a loved one’s death as related to pain control, comfort and joy as well as those aspects important to many patients such as having control over the circumstances of their death.	Supplemental
**Quality of Family Experience (at EOL)** ^44^	Quality of End-of-life Care	17		Measure of family members quality of experience of EoL care	Supplemental
**Quality of End of Life Care (QEOLC)** ^45^	Quality of End-of-life Care	10		QEOLC is an instrument in which respondents rate a clinician’s skill at providing high quality end-of-life care	Supplemental

**Table 5:** Psychological Wellbeing of Family and Surrogates

CDE/Scale Name	Subgroup	Number of Items/Questions	NINDS CDE ID	Brief Description	Classification
**Beck Depression Inventory (BDI-II)** ^46–49^	Surrogate Psychological distress	21	C20421+	Measures severity of depressive symptoms	Supplemental
**Beck Anxiety Inventory (BAI)** ^50,51^	Surrogate Psychological distress	21	21811, 21823–21868	Measure of anxiety	Supplemental
**Caregiver burden scale** ^52,53^	Surrogate Psychological distress	22	C11583	Assesses subjectively experienced burden by caregiver’s to chronically disabled persons	Supplemental
**Center for Epidemiologic Studies Depression Scale (CES-D)** ^54–58^	Surrogate Psychological distress	20	372	Measures depressive feelings and behaviors	Supplemental
**General Anxiety Disorder (GAD-7)** ^59–60^	Surrogate Psychological distress	7	C13205+	Measure anxiety disorders including post traumatic stress.	Supplemental
**Hospital Anxiety and Depression Scale (HADS)** ^61–63^	Surrogate Psychological distress	14		Assesses depression and anxiety	Supplemental
**Impact of Event Scale** ^64^	Surrogate Psychological distress	15	SCI	Screens for symptoms of avoidance and intrusion related to particular event	Supplemental
**Multidimensional Scale of Perceived Social Support (MSPSS)** ^65,66^	Surrogate Psychological distress	12	SCI	Assesses perception of social support.	Supplemental
**Patient Health Questionnaire** (PhQ-9)^67,68^	Surrogate Psychological distress	9	C07430+	Measures depression severity	Supplemental
**Zarit Caregiver Burden Scale** ^69^	Surrogate Psychological distress	29		Measure feelings of burden experienced by the caregivers of elderly persons with dementia. Caregivers are asked to respond to questions about the impact of the patients disabilities on their life	Supplemental
**Brief COPE Inventory** ^70^	Surrogate Psychological distress	28		Multidimensional measure of strategies for coping with stressors	Exploratory
**Brief Assessment Scale for Caregivers (BASC) of the Medically Ill** ^71^	Surrogate Psychological distress	14		Assessment of caregiver burden in the medically ill; has other cultural/language variants with additional questions	Exploratory
**Family strain questionnaire** ^72–75^	Surrogate Psychological distress	21–44		Self-reported strain of family caregivers	Exploratory
**Modified caregiver strain index (modified CSI)** ^26–31^	Surrogate Psychological distress	13		Assessment of strain on long-term caregiver	Exploratory
**National stressful events survey PTSD short scale (NSESSS)** ^76^	Surrogate Psychological distress	9		Dimensional assessment of PTSD	Exploratory
**PTSD checklist (PCL-5)** ^77,78^	Surrogate Psychological distress	20		Checklist for DSM criteria of PTSD	Exploratory
**ENRICHD Social Support Inventory (ESSI): mental health, assess social support** ^79–80^	Surrogate Psychological distress	7		Range of participant’s social support, multidimensional	Exploratory
**Prolonged grief revised scale (PG-13-R)** ^81^	Surrogate Psychological distress	13		Assessment of prolonged grief syndrome in family members of a decedent	Exploratory
